# A rare case of early onset lewy body dementia with parkinsonism associated with chronic exposure to copper contaminated drinking water

**DOI:** 10.3389/ftox.2024.1451235

**Published:** 2024-09-02

**Authors:** Marcia H. Ratner, Jonathan S. Rutchik

**Affiliations:** ^1^ Department of Pharmacology, Physiology and Biophysics, Boston University Chobanian & Avedisian School of Medicine, Boston, MA, United States; ^2^ Neurology, Environmental and Occupational Medicine Associates, CA and Division of Medicine, Occupational Medicine, University of California at San Francisco, San Francisco, CA, United States

**Keywords:** copper, α-synuclein, Parkinson’s disease, dementia with lewy bodies, water

## Abstract

There is a well-recognized relationship between a person’s body burden of essential trace elements such as copper and their neurological function in which both deficiencies and exposures to excessive concentrations are associated with adverse clinical outcomes. Preclinical studies indicate chronic excess copper exposure is associated with altered motor function, dopaminergic neuronal loss, astrocytosis, and microgliosis. Copper also promotes oligomerization and fibrilization of α-synuclein suggesting it may hasten the course of an α-synucleinopathy. Here we report a rare case of early onset Lewy Body Dementia with Parkinsonism in a 53-year-old Caucasian woman exposed to copper contaminated drinking water for more than 10 years. Her hair and that of her daughter had streaks of blue-green discoloration as did the porcelain sinks in their home. Testing confirmed copper contamination of the drinking water. A neurologist diagnosed her with Lewy Body Dementia with Parkinsonism. Skin biopsy for phosphorylated α was consistent with a diagnosis of an α-synucleinopathy. These findings suggest chronic exposure to excessive copper may act as disease modifying factor in Lewy Body Dementia with Parkinsonism. It has previously been recommended that individuals at risk of Alzheimer’s disease (AD) avoid excessive intake of copper. Genetic studies indicate that Lewy Body Dementia shares risk factors and pathways with AD. Based on the observations in this patient we recommend that individuals at risk for an α-synucleinopathy based on a positive family history, genetic testing, and/or positive results on a skin biopsy for phosphorylated α-synuclein avoid exposure to excess copper.

## Background

There is a well-recognized relationship between an individual’s body burden of essential trace elements such as iron, copper, and manganese and their neurological function in which both deficiencies and excessive exposure levels are associated with adverse clinical outcomes (Dusek et al., 2015; Ajsuvakova et al., 2020). Disrupted copper homeostasis has been shown to increase oxidative stress, neuroinflammation and aggregation of neurotoxic proteins such as α-synuclein, beta amyloid (Aβ) and tau implicated in neurodegenerative diseases (Brewer, 2008; Kitazawa et al., 2009; Lim et al., 2020; Wei et al., 2024). The role of environmental factors in the early-onset form of Dementia with Lewy Bodies (DLB) is poorly understood. The relationship between excessive copper exposure and risk for DLB has not been explored. This case report describes the clinical findings and exposure history of a 53 woman who developed symptoms of early onset DLB following a well-documented history of chronic exposure to copper via her consumption of contaminated drinking water.

## Case report

**FIGURE 1:**
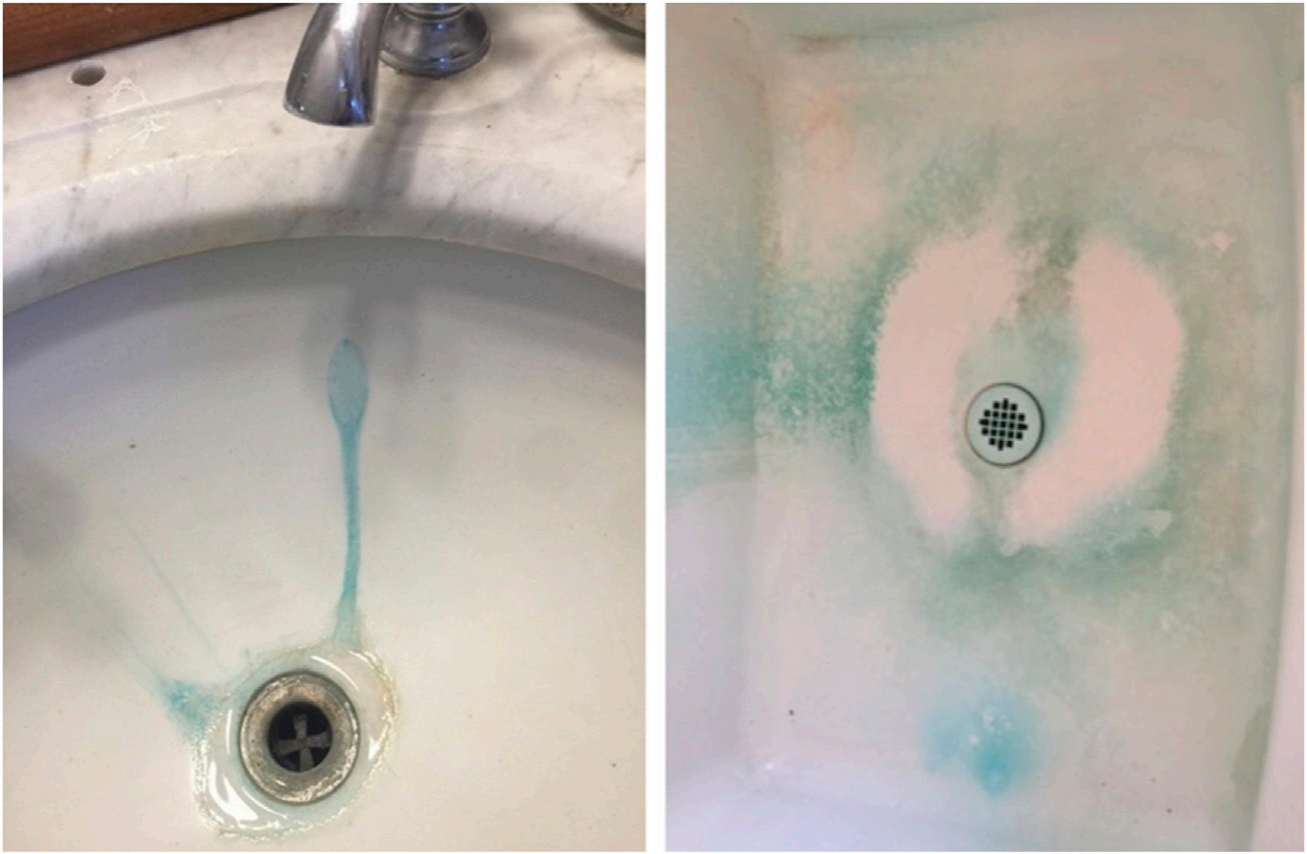
Blue-green stains due to copper contaminaton of water is seen on the actual sinks from the home.

Upon moving into a new residence in 2009 a 48-year-old right-handed, Caucasian woman with a history of bipolar disorder and her family members immediately noticed a metallic taste to the tap water. They initially attributed the metallic taste to the water being from a well rather than from a municipal water supply which they were more accustomed to. Shortly thereafter, the woman noticed that her and her daughter’s hair both had streaks of blue-green discoloration (Schwartz et al., 2014). Within a few months the blue-green discoloration in her daughter’s hair was so pronounced that she was admonished by a teacher for “coloring her hair” (the school she attended had a strict no-hair-dye policy). The family members also noted that the water was causing blue-green stains in their porcelain sinks (Figure 1). These problems with the metallic taste, hair discoloration and stains on the sinks continued until the family moved out of the residence in November of 2021.

In 2015, when the woman was just 53 years old, her family members noticed that she had a hand tremor. Around this time, the woman also began to experience mild cognitive problems. She consulted with her primary care physician about these new symptoms and was referred to a neurologist. Because she was taking lithium, the tremor which had an intentional component, was initially attributed to this medication. When her tremor did not resolve with discontinuation of lithium this etiology was subsequently ruled out. By 2018, her cognitive symptoms began to interfere with her ability work. She also had trouble passing the annual training sessions required for her to maintain the certifications she needed to keep her job.

In early April of 2021, the family was warned by a plumber about drinking copper contaminated water. Based on his warning they began using bottled water and water filtered through a Brita type water filter. A water analysis performed on 4/12/21 revealed a copper concentration of 3,100 μg/L (Table 1). This concentration exceeded the California Water Board maximum contamination levels (MCL) for copper in drinking water 1,000 μg/L (US EPA MCL goal/action level is 1,300 μg/L). Low pH water can cause gradual leaching of copper from pipes resulting in elevated levels in drinking water (Sonon et al., 2006). Independent inspection of plumbing dated 8/21/21 determined that the elevated copper was due to deterioration and oxidation of the copper pipes in the home. Water testing performed in 2017 also revealed an elevated copper concentration (2,300 μg/L). Unfortunately, this information which, could have reduced the total exposure to copper in this case, was not shared with the family until after the testing in April 2021 had been performed. Copper-contamination of domestic water supplies is associated with a metallic taste, blue-green stains on sinks and discoloration of hair (Sonon et al., 2006; Schwartz et al., 2014). However, because the family members were also unaware of these relationships, they took no action to reduce their exposure to copper.

**TABLE 1:** Results of laboratory analysis of water for toxic metals showing elevated copper levels.

Analysis	Results	Units	Date	MDL	MCL	Method
Arsenic	ND	μg/L	4/12/2021	1.67	10 μg/L	EPA 200.8
Chromium	ND	μg/L	4/12/2021	0.28	50 μg/L	EPA 200.7
Lead	ND	μg/L	4/12/2021	1.4	15 μg/L	EPA 200.8
Copper	3100	μg/L	4/12/2021	0.28	1,000 μg/L	EPA 200.7

ND, not detected; MDL, method detection limit; MCL, maximum contaminant level.

The woman reported drinking up to a gallon of tap water per day. Assuming she consumed a more conservative average of 2.7 L of tap water daily, this would result in an average intake of 8,370 μg/day (8.37 mg/day) during the 10 years she was exposed to copper. It is important to note that this estimate does not include additional copper intake from food sources. Therefore, her total daily intake of copper would be expected to be even higher. None of the family members recalled experiencing any specific gastrointestinal (GI) symptoms while exposed to copper at these concentrations. However, studies indicate that not all persons exposed to copper experience GI symptoms (Araya et al., 2001; 2003). The woman had no history of iron deficiency or anemia. It is important to note that the estimates of exposure used in this case report are necessary because there are currently no biomarkers that accurately and reliably assess copper status. As a result, copper status is not routinely assessed in clinical practice (Prohaska, 2012).

The woman underwent neuropsychological testing in December of 2021. This assessment revealed impaired performance on tests of verbal and visual memory function. Performance on tests of attention and executive functioning were also below expectation (McKeith et al., 2017). Self-report measures of mood indicated moderate symptoms of depression and mild anxiety. A skin biopsy test for phosphorylated α-synuclein performed in October of 2022 was positive. This finding is consistent with diagnosis of an α-synucleinopathy such as DLB (Gibbons et al., 2024). A DaTscan performed in June of 2022 was not possible to interpret due to movement artifacts. A subsequent DaTscan performed in July of 2022 was interpreted as normal. An MRI could not be performed because of a cochlear implant. A CT scan was therefore performed. The CT scan was interpreted as normal showing no indication of medial temporal lobe atrophy as would be seen in AD. Treatment with carbidopa/levodopa alleviated her motor symptoms (Molloy et al., 2005).

The woman subsequently contacted a neurotoxicologist (MHR) in March of 2023 with concerns about the possible relationship between the relatively early onset of her neurological symptoms and her history of chronic exposure to excessive copper. The neurotoxicologist thoroughly reviewed her history and symptoms with a board-certified neurologist (JSR) with additional expertise in occupational and environmental medicine. The neurotoxicologist also referred the woman to a board-certified neurologist with expertise in the diagnosis of Parkinson’s disease and parkinsonism due to exposure to neurotoxic metals for an additional opinion. The results of this independent neurological examination which was performed in August of 2023 revealed findings consistent with a diagnosis of Lewy Body Dementia with parkinsonism. The neurologist noted that it was not possible to obtain biospecimens to confirm her copper exposure because this had ceased more than a year prior to his examination. He also noted that chelation therapy would not be of any benefit to her at that time since her exposure to the source of copper had ended and thus, any excessive body burden of would have already been eliminated.

## Discussion

Copper is an essential trace element necessary to normal biological functions. Copper is carried in the blood by ceruloplasmin. Dietary intake from food sources is the primary source of copper exposure in adults. Concentrations of total (free and bound) serum copper range between 64 and 140 μg/dL. The U.S. Food and Drug Administration’s recommended daily allowance for copper is 0.9 mg (900 μg) for adults. According to the NIH Office of Dietary Supplements Fact Sheet for Health Professionals, the average daily intake of copper from dietary sources for an adult woman is 1,100 μg/day. The adult daily upper limit for copper from all sources (food, beverages, and supplements) 10,000 ug (10 mg). These data suggest that the woman was at risk for daily exposures to copper at or above the recommended upper daily exposure limit.

Gastrointestinal symptoms are frequently reported among otherwise healthy subjects exposed to copper via contaminated drinking water including copper leaching from copper plumbing pipes (Spitalny et al., 1984; Knobeloch et al., 1994; Eife et al., 1999; Pizarro et al., 1999; 2001; Stenhammar 1999; Araya et al., 2003). A study looking at chronic copper exposure found that 19/340 subjects reported at least one gastrointestinal (GI) symptom on one occasion during 2 months of exposure to 6 mg/L indicating that most persons exposed to excess copper do not experience any GI symptoms (Araya et al., 2003). The subjects also had normal liver function tests during exposure to this same concentration of copper (Araya et al., 2003). Based on research in an apparently healthy population of 179 individuals in which nausea was the most frequently report acute symptom the No-Observed-Adverse-Effect-Level and Lowest-Observed-Adverse-Effect Level were determined to be 4 and 6 mg Cu/L (0.8 and 1.2 mg Cu) respectively (Araya et al., 2001). A study by Pizarro and colleagues (2001) looked at gastrointestinal symptoms in 45 healthy adult women ages 18–55 years-old who ingested tap water with 5 mg/L of copper over a 9-week period. Less than half (20/45) of these subjects reportedly experienced gastrointestinal disturbances at least once during the study. Among these subjects 9/45 reportedly experience diarrhea either with or without abdominal pain and vomiting, and the other 11/45 subjects reported abdominal pain, nausea, or vomiting (Pizarro et al., 2001). Gastrointestinal symptoms have been shown to resolve with cessation of exposure (Spitalny et al., 1984; Stenhammar 1999).

Both copper deficiency and copper overload have been associated with an increased risk for neurodegenerative disease including Parkinson’s (PD) (Ajsuvakova et al., 2020; Bisaglia and Bubacco, 2020). Wilson’s disease (WD) is an example of a specific well-studied inherited disease associated a mutation in the ATPase copper transporting beta gene which encodes for a protein responsible for removing extra copper from the body (Dusek et al., 2015). This protein facilitates the efflux of copper from the liver into the bile. Copper is primarily excreted in bile with a smaller amount excreted in the urine. Patients with Wilson’s disease have an increased risk for developing Parkinson’s disease (Ortiz et al., 2020; Johnson, 2001; Palmieri et al., 2022). Studies of patients with Wilson’s disease indicate that excess copper accumulates equally in different parts of the brain (Litwin et al., 2013).

The α-synucleinopathies include PD, DLB, and multiple system atrophy. Among these, PD is the most well-studied. Aside from genetics, age is the main risk factor for PD (de Lau and Breteler, 2006; Pringsheim et al., 2014). A meta-analysis of worldwide prevalence data performed by Pringsheim and colleagues (2014) revealed that the prevalence of PD increases with age from 41 per 100,000 subjects between the ages of 40–49 years, to 1,903 per 100,000 in subjects older than age 80. The prevalence of PD is higher in men than women (Pringsheim et al., 2014). The incidence of PD before age 50 years-old was found to be 0.81/100,000 person-years (1.98 in Parkinsonism all type) and prior age 55 years was 2.05/100,000 person-years (5.05 for Parkinsonism of all types) with a higher incidence seen in men than women (Camerucci et al., 2021). Early onset of PD is seen among subjects exposed to neurotoxicants (Ratner et al., 2014; Gamache et al., 2019).

The pathogenesis of PD is complex involving convergence of genetic and environmental risk factors which share mechanisms of action (Tysnes and Storstein, 2017; Simon et al., 2020; Ben-Shlomo et al., 2024). These mechanisms include disrupting mitochondrial function, promoting aggregation of α-synuclein and increasing oxidative stress (Tysnes and Storstein, 2017; Simon et al., 2020). Occupational and environmental exposures implicated in PD include pesticides and herbicides (e.g., paraquat), solvents (e.g., trichloroethylene) and metals (e.g., manganese) (Ratner et al., 2014; Gamache et al., 2019; Adamson et al., 2023; Goldman et al., 2023; Newell et al., 2024). Although neurotoxicants such as copper may not cause PD these compounds can exacerbate the actions of other factors involved in the pathogenesis of this disease such as oxidative stress (Cruces-Sande et al., 2019). It has recently been reported that exposure to trichloroethylene and other volatile organic compounds decades earlier is associated a more rapid progression of PD symptoms (Goldman et al., 2024).

Unlike patients with PD who typically present with a resting tremor, patients with DLB often present with cognitive deficits and a complex pattern of mixed tremors, characterized by rest and postural/action tremors (Onofrj et al., 2013). Genome-wide association analysis indicates that DLB shares risk profiles and pathways with Alzheimer’s disease (AD) and PD (Chia et al., 2021). A retrospective study by Sim and colleagues (2022) determined that the mean age at onset of patients with the early onset form of DLB is 57.9 years old. The same study found that the time from diagnosis to death was 3.3 years in the patients with early onset DLB consistent with previous reports showing average survival time from diagnosis to death is just 4.11 years. There were only nine female subjects in this small study which, also did not control for history of occupational or environmental exposures to neurotoxic chemicals (e.g., metals) as a risk factor for the early onset of form of DLB. A recent review of the literature suggests that exposure to metals including copper may play a role in risk for Lewy body disease (Boström et al., 2009; An and Xu, 2024).

Other than genetics and age, the causes of age-related neurodegenerative diseases such as PD, DLB and AD have not been fully elucidated. Nevertheless, aggregation of endogenous proteins such as α-synuclein and environmental factors including exposure to metals are widely held to be involved (Gamache et al., 2019; Gonzalez-Alcocer et al., 2023). Disrupted homeostasis of copper has been shown to increase oxidative stress, promote aggregation of neurotoxic proteins implicated in neurodegenerative diseases including α-synuclein, Aβ, and tau and, to increase neuroinflammation (Brewer, 2008; Kitazawa et al., 2009; Dell'Acqua et al., 2015; Lim et al., 2020; Patel and Aschner, 2021). Ajsuvakova et al. (2020) reported that the copper to ceruloplasmin ratio was reduced in PD patients indicating that copper ions were not bound to ceruloplasmin as expected, but on the contrary copper was either free or bound to other low molecular weight species such as amino acids. Free Cu contributes to oxidative stress and binds to α-synuclein leading to neuronal degeneration. Increased concentrations of copper have been observed in the cerebrospinal fluid of Parkinson’s patients (Pall et al., 1987; Davies et al., 2016).

Studies of chronic copper exposure using a physiologically relevant model were found to alter motor function and induce dopaminergic neuronal loss, astrocytosis, and microgliosis in a dose-dependent manner in (Gonzalez-Alcocer et al., 2023). Copper exposure was also associated with a concentration-dependent increase in nitrosative stress. Accumulation and aggregation of α-synuclein were both increased. Copper has previously been shown to promote α-synuclein oligomerization and fibrilization (Paik et al., 1999; Uversky et al., 2001; Ajsuvakova et al., 2020; Li et al., 2022; Synhaivska et al., 2022; Savva and Platts, 2024). This observation is important because it provides a mechanism via which this metal can contribute to the progression of α-synucleinopathies such as PD and DLB by promoting formation of oligomers which are more stable and less prone to dissociation than aggregates formed in the metal-free systems (Savva and Platts, 2024). The physiological form of α-synuclein is N-terminally acetylated. Some researchers have cautioned about overinterpreting work that predates the use of the acetylated form of this protein. Miotto and colleagues (2015) demonstrated that the formation of an acetylated α-synuclein copper complex at the N-terminal region stabilizes local conformations with α-helical secondary structure and restricted motility (Miotto et al., 2015). Subsequent work by Teng et al. (2021) showed that acetylated α-synuclein displays several orders of magnitude weaker copper binding affinity than wild-type α-synuclein. These findings indicate that this interaction requires exposure to physiologically excessive concentrations of copper such as occurs during exposure to copper contaminated drinking water. Public drinking water supplies generally have copper levels well below 3,100 μg/L (Fewtrell et al., 1996). However, the concentration of copper in the water supply in the current case report was found to be three times higher than the California Water Board’s MCL level (3100 μg/L versus MCL of 1000 μg/L). The sink stains, hair discoloration and metallic taste experienced by the family members indicate that contaminated tap water was a source of chronic copper exposure for over 10 years (Gonzalez-Alcocer et al., 2023).

It has been suggested that individuals at increased risk of AD avoid excessive intake of copper. However, no such recommendation has yet to be made for individuals at risk for α-synucleinopathies such as PD or DLB (Barnard et al., 2014). Many cases of DLB also show AD neuropathology in the form of amyloid-β plaques and tau neurofibrillary tangles. Copper has also been implicated in dementia of the Alzheimer’s type. *In vitro* studies indicate that the inorganic cupric ion (Cu^2+^) which is similar to the copper that leaches from copper water pipes into tap water supplies potentiates Aβ neurotoxicity in cell cultures (Huang et al., 1999). Exposure to copper contaminated drinking water has also been shown to accelerate amyloid and tau neuropathology in a mouse model of AD (Kitazawa et al., 2009). Copper exposure upregulates the degenerative genes and represses homeostatic genes within microglia even in the absence Aβ plaques suggesting that excess copper exposure alone perturbs microglial homeostasis and contributes to accelerated cognitive decline (Lim et al., 2020).

Alternative etiologies were also considered in this case. The tremor associated with lithium typically appears when treatment with the drug is initiated or when the patient is being titrated and decreases over time with ongoing treatment. The differential diagnosis of tremor in patients on lithium includes metabolic abnormalities, benign essential tremor, PD, and lithium toxicity (Baek et al., 2014). Reducing the dose of lithium is helpful in many cases. Lithium also has a narrow therapeutic window and exposure to excess lithium is associated with the “syndrome of irreversible lithium-effectuated neurotoxicity (SILENT). This syndrome typically follows an identifiable lithium overdose (Schou, 1984; Farouji et al., 2023). Acute overdose on top of chronic exposure (acute on chronic) is associated with greatest risk of irreversible neurological consequences due to lithium. The putative cause of SILENT is demyelination caused by lithium at multiple sites within the nervous system. There were no reports of any acute on chronic overdoses in the woman described in this case report and this was ruled out as an etiology of her symptoms. In addition, the skin biopsy showing phosphorylated α-synuclein supports a diagnosis of an α-synucleinopathy such as DLB in this case.

Although her DaTscan was interpreted as normal it is important to note that this case report involves chronic exposure to excessive copper and, that no studies to date have explored the relationship between DaTscan findings in early DLB onset in persons with a history of exposures to neurotoxicants. The CT scan which failed to show evidence of medial temporal lobe atrophy, the attention and executive function deficits on neuropsychological testing, the response to dopaminergic therapeutics, and the positive skin biopsy showing phosphorylated α-synuclein all supported a diagnosis of DLB and were therefore used to differentiate the findings in this case from those due to AD (McKeith et al., 2017).

## Conclusion

This rare case report of a well-documented history of excessive copper exposure from a contaminated home water supply, and the comprehensive review of the relevant peer-reviewed literature cited herein, supports the conclusion that exposure to copper that leached from plumbing pipes served as a disease-modifying factor that contributed to the age at onset of symptoms of Lewy Body Dementia with parkinsonism observed in this case report. The slow clinical course of the disease from onset to the present time, and the well-documented exposure history to a metal known to promote aggregation of α-synuclein indicate that this case is not a typical example of early onset DLB (Mueller et al., 2019; Sim et al., 2022). These findings should not be interpreted to suggest that exposure to copper causes DLB or PD *per se* (Cruces-Sande et al., 2019). However, these findings do provide data demonstrating that exposure to excessive concentrations of copper did occur in this case. This excessive exposure continued for over 10 years until an astute plumber warned the family about the risks of drinking copper contaminated water, which ultimately led to the water supply being tested for copper. The review of the literature included in this case report provides data from preclinical models demonstrating relevant interactions between copper and α-synuclein. These interactions have the potential to mechanistically exacerbate underlying age-related neurodegenerative disease processes such as protein aggregation in an at-risk individual which would be expected *a priori* to contribute to a younger age at disease onset (Li et al., 2022). Additional research is needed to further elucidate the associations between neurodegenerative diseases such as DLB and chronic exposure to excessive concentrations of copper via drinking water, especially when the exposure persists for many years. It has been suggested by other authors that individuals at increased risk of AD should avoid excessive intake of copper (Barnard et al., 2014). Based on the findings presented in this case report, the mechanistic literature cited herein, and the results of genetic studies indicating that DLB shares risk profiles and pathways with AD and PD, we now recommend that individuals at risk for α-synucleinopathies based on their family history or genetic testing avoid exposure to excess copper (Tsuang et al., 2002; Chia et al., 2021).
